# Associations between caffeine consumption, anxiety, temporomandibular disorders, and oral behaviors in university students in Brazil—a cross-sectional study

**DOI:** 10.22514/jofph.2026.061

**Published:** 2026-09-12

**Authors:** Ana Luiza Azevedo Gontijo, Monalisa Teixeira Santos de Macedo, Stefany Joaquina Sousa Farias, Edivar Ximenes de Aragao Junior, Karoline Bittencourt Mendes Leitão, Evelyn Mikaela Kogawa, Rodrigo Antonio de Medeiros

**Affiliations:** ^1^Department of Dentistry, School of Health Sciences, University of Brasília (UnB), 70910-900 Brasilia, Brazil

**Keywords:** Temporomandibular disorders, Anxiety, Caffeine, Students

## Abstract

**Background**: This cross-sectional study investigated the associations between caffeine consumption, caffeine-related expectations, anxiety symptoms, painful temporomandibular disorder (TMD) symptoms, and oral behaviors in university students. **Methods**: Students from the Faculty of Health Sciences at the University of Brasília completed a self-administered questionnaire assessing their sociodemographic characteristics, caffeine consumption frequency, expected effects of caffeine, anxiety symptoms, painful TMD symptoms, and oral behaviors. Associations between categorical variables were analyzed using the chi-square or Fisher’s exact tests, and correlations between caffeine consumption and expectancy domains were assessed using Spearman’s correlation coefficient. Statistical analyses were performed at a significance level of 5%. **Results**: A total of 182 students participated in this study. Painful TMD symptoms were significantly more prevalent among students with severe anxiety (*p* < 0.001). A higher prevalence was also observed among female participants and those who reported frequent oral behaviors. Oral behaviors were significantly associated with both anxiety symptoms and female sex (*p* < 0.001). No significant association was found between overall caffeine consumption and oral behaviors. However, daily black coffee consumption was significantly associated with severe anxiety (*p* < 0.001). Additionally, specific types of caffeinated products were positively correlated with distinct expectations about their effects, such as increased energy, dependence, and mood enhancement. **Conclusions**: Although overall caffeine consumption was not directly associated with oral behaviors or painful TMD symptoms, daily black coffee intake was associated with higher anxiety levels in this study sample. These findings suggest a possible association between black coffee consumption, anxiety symptoms, and painful TMD-related outcomes; although causality cannot be established because of the observational design of the study.

## 1. Introduction

Temporomandibular disorders (TMD) are multifactorial conditions that affect the masticatory muscles, temporomandibular joints (TMJs), and associated structures. Their clinical manifestations typically include orofacial pain, restricted jaw movement, joint sounds, and muscle tenderness [1]. Multiple factors have been linked to the development of TMD symptoms, including genetic predisposition, trauma, and psychosocial influences, particularly anxiety, oral behaviors, and bruxism-related activities [2]. These are currently understood as distinct masticatory muscle activities rather than parafunctional habits [3].

Systematic reviews indicate that temporomandibular disorders (TMD) affect a substantial proportion of the global population, with prevalence estimates varying according to the diagnostic criteria, age, sex, and geographic region studied [4, 5]. However, the prevalence appears to be even higher among university students, particularly those enrolled in health-related programs, with recent studies reporting a high frequency of TMD symptoms in this population [6, 7]. Furthermore, studies have found that students experience greater TMD symptomatology and associated psychosocial factors compared with the general population, reinforcing their vulnerability to developing TMD. This highlights the importance of investigating the behavioral and psychological factors related to TMD in university settings [8, 9].

University students may be especially vulnerable to TMD symptoms, anxiety, and reduced quality of life due to high levels of academic and social stress [10]. Concurrently, caffeine consumption is common in this population, largely because of its stimulating effects on the central nervous system. Common natural sources of caffeine include coffee, tea, guarana, cocoa, and mate. However, caffeine has been suggested to exacerbate anxiety, disrupt sleep, and potentially aggravate TMD-related symptoms [7].

Additionally, individual responses to caffeine can vary owing to physiological and behavioral factors. Caffeine exerts stimulating effects on the central nervous system and can influence mood, arousal, and behavioral responses [11, 12]. Caffeine has therefore been associated with increased anxiety and sleep disturbances, which may contribute to the exacerbation of TMD symptoms [7, 10, 13, 14].

For dental professionals, understanding the interplay between caffeine use, anxiety, oral behaviors, and TMD is essential for delivering comprehensive care. Although previous studies have explored the effects of caffeine on anxiety and pain perception, few have examined the simultaneous associations among caffeine consumption, anxiety symptoms, oral behaviors, and TMD manifestations within a single population [10]. Investigating these factors together may help clarify how behavioral and psychological characteristics interact in individuals with TMD symptoms, particularly in populations exposed to high-stress levels, such as university students. Furthermore, TMD comprises a broad group of conditions with both painful and nonpainful manifestations, most commonly assessed using the Diagnostic Criteria for Temporomandibular Disorders (DC/TMD) [15].

This study aimed to investigate the relationships among caffeine consumption, expectations regarding its effects, anxiety symptoms, oral behaviors, and temporomandibular disorder (TMD) symptoms in university students. We hypothesized that anxiety symptoms would be associated with oral behaviors and painful TMD symptoms and that caffeine consumption and caffeine-related expectations would be associated with anxiety symptoms.

## 2. Materials and methods

This cross-sectional study was conducted with students enrolled in the Dentistry, Nutrition, Nursing, Pharmacy, and Public Health programs at the Faculty of Health Sciences, University of Brasília (UnB), Brazil. Ethical approval was obtained from the National Research Ethics Committee (CAAE: 70165723.5.0000.0030).

The minimum sample size was calculated using OpenEpi software (version 2.3.15) for a student population of 1967, with a 95% confidence level, a 5% margin of error, and a design effect of 1.0, yielding a minimum of 179 participants. A total of 182 students participated in the study.

The inclusion criteria were an age of ≥18 years, current enrollment in one of the listed programs, and the provision of informed consent. Participants who submitted incomplete or improperly completed questionnaires were excluded from the study. Cases with missing data were handled using listwise exclusion, depending on the analysis performed.

Data were collected between September 2023 and January 2024. Students were invited to participate through Quick Response (QR) codes and social media using a convenience sampling approach, and their responses were obtained via a self-administered online questionnaire. Participation was voluntary and anonymous, and no identifying information was collected from participants. Measures were implemented to prevent duplicate responses by restricting multiple submissions from the same device. Owing to the convenience sampling strategy and voluntary participation, selection bias may have occurred, resulting in a higher proportion of female participants. The instrument was divided into six sections: (1) sociodemographic information (age, gender, and academic program), (2) caffeine consumption habits, (3) expectations regarding the effects of caffeine, (4) anxiety symptoms, (5) TMD symptoms, and (6) oral behaviors.

Caffeine consumption was assessed using participants’ self-reported frequency of intake of caffeine-containing products, including black coffee, mate tea, cola beverages, energy drinks, and dark chocolate. Participants reported their habitual consumption patterns using predefined frequency categories. Because no standardized and validated instrument was available for quantifying caffeine intake in this context, no conversion to total daily caffeine intake (mg) was performed. Instead, the analysis focused on the consumption patterns of specific caffeine sources.

Expectations regarding the effects of caffeine were assessed using the Brief Version of the Caffeine Expectancy Questionnaire for Brazil (B-CaffEQ-BR) questionnaire [16], a brief, validated Brazilian adaptation of the Caffeine Expectancy Questionnaire (CEQ). This tool includes 21 items across seven domains: dependence, energy/performance, appetite suppression, mood/socialization, physical performance, anxiety/negative effects, and sleep disturbance. Responses to the B-CaffEQ-BR questionnaire were recorded on a six-point Likert scale, and mean scores were computed for each domain of the questionnaire.

TMD symptoms were identified using the Portuguese-translated version of the DC/TMD Symptom Questionnaire [15, 17]. Based on self-reported symptoms within the past 30 days, participants were categorized into three groups: no TMD, painful TMD (including orofacial pain and headache), and nonpainful TMD (such as clicking or jaw locking).

Anxiety symptoms were evaluated using the Portuguese-validated Generalized Anxiety Disorder-7 (GAD-7) scale [9], as recommended by the DC/TMD guidelines. Scores ranged from 0 to 21, with cutoff scores of 5, 10, and 15 indicating mild, moderate, and severe anxiety, respectively.

Oral behaviors were assessed using the Oral Behaviors Checklist (OBC) [15], which quantifies the frequency of awake and sleep-related oral habits. Scores were classified into three categories according to the DC/TMD manual [18]: no behavior (score = 0), low frequency (scores 1–24), and high frequency (scores 25–84).

The data were tabulated and analyzed using Jamovi software. Associations between categorical variables were examined using the chi-square and Fisher’s exact tests, whereas Spearman’s correlation coefficient was applied to assess the relationships between the frequency of caffeine consumption and expectancy domains. The significance level was set at *p* < 0.05. Given the cross-sectional and self-reported nature of the study, potential sources of bias include selection bias, recall bias, and the inability to establish causal relationships.

## 3. Results

A total of 182 students participated in the study, of whom the majority were female (78%). Most participants were enrolled in Dentistry (41.8%), followed by Nursing (19.2%), Nutrition (17.6%), Pharmacy (17%), and Public Health (4.4%) programs. This distribution reflects the composition of undergraduate programs at the Faculty of Health Sciences.

Regarding anxiety symptoms, 20.9% of the participants reported no symptoms, 24.7% had mild symptoms, 26.9% had moderate symptoms, and 27.5% had severe symptoms. Thus, more than half of the sample reported moderate-to-severe anxiety symptoms.

As shown in Table 1, painful TMD symptoms were significantly associated with higher anxiety levels (*p* < 0.001), female sex (*p* < 0.001), and more frequent oral behaviors (*p* < 0.001). Students with severe anxiety, female participants, and those who reported high-frequency oral behaviors were more likely to report painful TMD symptoms. These associations were identified using chi-square tests and demonstrated consistent relationships between psychosocial and behavioral factors and painful TMD symptoms.

**Table 1.** Frequency and percentage of study participants according to the association between TMD symptoms and different variables (Chi-square and Fisher’s exact test, *p* < 0.05).

Variables		TMD Symptoms			Statistical Test	*p*-value
		No TMD	Non-painful TMD	Painful TMD		
Anxiety Symptoms						
	None	17 (44.7%)	8 (21.1%)	13 (34.2%)	χ^2^	<0.001*
	Mild	11 (24.4%)	5 (11.1%)	29 (64.4%)		
	Moderate	7 (14.3%)	10 (20.4%)	32 (65.3%)		
	Severe	4 (8.0%)	6 (12.0%)	40 (80.0%)		
Oral Behaviors						
	None	1 (100%)	0 (0%)	0 (0%)	Fisher	<0.001*
	Low	34 (33.3%)	23 (22.5%)	45 (44.1%)		
	High	4 (5.1%)	6 (7.6%)	69 (87.3%)		
Course						
	Nursing	6 (17.1%)	2 (5.7%)	27 (77.1%)	Fisher	0.443
	Pharmacy	5 (16.1%)	5 (16.1%)	21 (67.7%)		
	Nutrition	10 (31.3%)	6 (18.8%)	16 (50.0%)		
	Dentistry	17 (22.4%)	14 (18.4%)	45 (59.2%)		
	Public Health	1 (12.5%)	2 (25.0%)	5 (62.5%)		
Gender						
	Female	21 (14.8%)	20 (14.1%)	101 (71.1%)	χ^2^	<0.001*
	Male	18 (45.0%)	9 (22.5%)	13 (32.5%)		

*: p < 0.05. χ^2^: Chi-square test. TMD: temporomandibular disorders.

Table 2 shows a significant association between oral behaviors and anxiety levels (*p* < 0.001), with more frequent behaviors observed among individuals with severe anxiety. Gender was also significantly associated with oral behaviors (*p* < 0.001), with a higher prevalence among female participants. The direction of these associations suggests that the frequency of oral behavior increased with increasing anxiety severity.

**Table 2.** Frequency and percentage of study participants according to the association between oral behaviors and different variables (Fisher’s exact test, *p* < 0.05).

Variables		Oral Behaviors			*p*-value
		None	Low	High	
Anxiety Symptoms					
	None	1 (2.6%)	32 (84.2%)	5 (13.2%)	<0.001*
	Mild	0 (0%)	27 (60.0%)	18 (40.0%)	
	Moderate	0 (0%)	26 (53.1%)	23 (46.9%)	
	Severe	0 (0%)	17 (34.0%)	33 (66.0%)	
Course					
	Nursing	0 (0%)	18 (51.4%)	17 (48.6%)	0.665
	Pharmacy	0 (0%)	16 (51.6%)	15 (48.4%)	
	Nutrition	1 (3.1%)	19 (59.4%)	12 (37.5%)	
	Dentistry	0 (0%)	44 (57.9%)	32 (42.1%)	
	Public Health	0 (0%)	5 (62.5%)	3 (37.5%)	
Gender					
	Female	1 (0.7%)	69 (48.6%)	72 (50.7%)	<0.001*
	Male	0 (0%)	33 (82.5%)	7 (17.5%)	

*: p < 0.05.

No significant associations were found between caffeine consumption frequency and TMD symptoms (Table 3) or oral behaviors (Table 4). However, daily black coffee consumption was significantly associated with the severity of anxiety symptoms (*p* < 0.001; Table 5); students with severe anxiety were more likely to consume black coffee daily. This finding highlights a specific relationship between black coffee consumption and anxiety severity, rather than with TMD symptoms or oral behaviors.

**Table 3.** Frequency and percentage of study participants according to the association between TMD symptoms and caffeine consumption frequency (Fisher’s exact test, *p* < 0.05).

Food	Frequency	TMD Symptoms			*p*-value
		No TMD	Non-painful TMD	Painful TMD	
Black Coffee					
	Never consume	16 (21.3%)	16 (21.3%)	43 (57.3%)	0.437
	Use once a week	5 (33.3%)	1 (6.7%)	9 (60.0%)	
	Use two to three times a week	4 (28.6%)	1 (7.1%)	9 (64.3%)	
	Use three or more times a week	5 (22.7%)	5 (22.7%)	12 (54.5%)	
	Use every day	9 (16.1%)	6 (10.7%)	41 (73.2%)	
Mate tea					
	Never consume	27 (18.6%)	27 (18.6%)	91 (62.8%)	0.307
	Use once a week	6 (28.6%)	2 (9.5%)	13 (61.9%)	
	Use two to three times a week	3 (50.0%)	0 (0%)	3 (50.0%)	
	Use three or more times a week	3 (30.0%)	0 (0%)	7 (70.0%)	
	Use every day	0 (0%)	0 (0%)	0 (0%)	
Coca-Cola					
	Never consume	13 (23.6%)	9 (16.4%)	33 (60.0%)	0.587
	Use once a week	13 (18.1%)	12 (16.7%)	47 (65.3%)	
	Use two to three times a week	9 (29.0%)	6 (19.4%)	16 (51.6%)	
	Use three or more times a week	2 (11.1%)	1 (5.6%)	15 (83.3%)	
	Use every day	2 (33.3%)	1 (16.7%)	3 (50.0%)	
Energy Drinks					
	Never consume	30 (22.9%)	20 (15.3%)	81 (61.8%)	0.757
	Use once a week	8 (22.2%)	8 (22.2%)	20 (55.6%)	
	Use two to three times a week	0 (0%)	1 (12.5%)	7 (87.5%)	
	Use three or more times a week	1 (20.0%)	0 (0%)	4 (80.0%)	
	Use every day	0 (0%)	0 (0%)	2 (100%)	
Dark Chocolate					
	Never consume	14 (27.5%)	7 (13.7%)	30 (58.8%)	0.492
	Use once a week	16 (21.3%)	13 (17.3%)	46 (61.3%)	
	Use two to three times a week	5 (13.2%)	9 (23.7%)	24 (63.2%)	
	Use three or more times a week	3 (21.4%)	0 (0%)	11 (78.6%)	
	Use every day	1 (25.0%)	0 (0%)	3 (75.0%)	

*TMD: temporomandibular disorders.*

**Table 4.** Frequency and percentage of study participants according to the association between oral behaviors and caffeine consumption frequency (Fisher’s exact test, *p* < 0.05).

Food	Frequency	Oral Behaviors			*p*-value
		None	Low	High	
Black Coffee					
	Never consume	0 (0%)	48 (64.0%)	27 (36.0%)	0.139
	Use once a week	0 (0%)	10 (66.7%)	5 (33.3%)	
	Use two to three times a week	0 (0%)	7 (50.0%)	7 (50.0%)	
	Use three or more times a week	1 (4.5%)	12 (52.5%)	9 (40.9%)	
	Use every day	0 (0%)	25 (44.6%)	31 (55.4%)	
Mate tea					
	Never consume	0 (0%)	78 (53.8%)	67 (46.2%)	0.251
	Use once a week	1 (4.8%)	13 (61.9%)	7 (33.3%)	
	Use two to three times a week	0 (0%)	4 (66.7%)	2 (33.3%)	
	Use three or more times a week	0 (0%)	7 (70.0%)	3 (30.0%)	
	Use every day	0 (0%)	0 (0%)	0 (0%)	
Coca-Cola					
	Never Consume	1 (1.8%)	32 (58.2%)	22 (40.0%)	0.942
	Use once a week	0 (0%)	41 (56.9%)	31 (43.1%)	
	Use two to three times a week	0 (0%)	16 (51.6%)	15 (48.4%)	
	Use three or more times a week	0 (0%)	10 (55.6%)	8 (44.4%)	
	Use every day	0 (0%)	3 (50.0%)	3 (50.0%)	
Energy Drinks					
	Never consume	1 (0.8%)	75 (57.3%)	55 (42.0%)	0.492
	Use once a week	0 (0%)	22 (61.1%)	14 (38.9%)	
	Use two to three times a week	0 (0%)	3 (37.5%)	5 (62.5%)	
	Use three or more times a week	0 (0%)	2 (40.0%)	3 (60.0%)	
	Use every day	0 (0%)	0 (0%)	2 (100%)	
Dark Chocolate					
	Never consume	1 (2.0%)	25 (49.0%)	25 (49.0%)	0.733
	Use once a week	0 (0%)	42 (56.0%)	33 (44.0%)	
	Use two to three times a week	0 (0%)	24 (63.2%)	14 (36.8%)	
	Use three or more times a week	0 (0%)	8 (57.1%)	6 (42.9%)	
	Use every day	0 (0%)	3 (75.0%)	1 (25.0%)	

**Table 5.** Frequency and percentage of study participants according to the association between anxiety symptoms and caffeine consumption frequency (Fisher’s exact test, *p* < 0.05).

Food	Frequency	Anxiety Symptoms				*p*-value
		None	Mild	Moderate	Severe	
Black Coffee						
	Never consume	21 (28.0%)	15 (20.0%)	22 (29.3%)	17 (22.7%)	<0.001*
	Use once a week	4 (26.7%)	4 (26.7%)	4 (26.7%)	3 (20.0%)	
	Use two to three times a week	0 (0%)	11 (78.6%)	1 (7.1%)	2 (14.3%)	
	Use three or more times a week	6 (27.3%)	3 (13.6%)	7 (31.8%)	6 (27.3%)	
	Use every day	7 (12.5%)	12 (21.4%)	15 (26.8%)	22 (39.3%)	
Mate tea						
	Never consume	27 (18.6%)	34 (23.4%)	41 (28.3%)	43 (29.7%)	0.557
	Use once a week	6 (28.6%)	5 (23.8%)	4 (19.0%)	6 (28.6%)	
	Use two to three times a week	2 (33.3%)	3 (50.0%)	1 (16.7%)	0 (0%)	
	Use three or more times a week	3 (30.0%)	3 (30.0%)	3 (30.0%)	1 (10.0%)	
	Use every day	0 (0%)	0 (0%)	0 (0%)	0 (0%)	
Coca-Cola						
	Never consume	15 (27.3%)	13 (23.6%)	8 (14.5%)	19 (34.5%)	0.067
	Use once a week	17 (23.6%)	16 (22.2%)	25 (34.7%)	14 (19.4%)	
	Use two to three times a week	5 (16.1%)	12 (38.7%)	6 (19.4%)	8 (25.8%)	
	Use three or more times a week	0 (0%)	3 (16.7%)	8 (44.4%)	7 (38.9%)	
	Use every day	1 (16.7%)	1 (16.7%)	2 (33.3%)	2 (33.3%)	
Energy Drinks						
	Never consume	29 (22.1%)	33 (25.2%)	34 (26%)	35 (26.7%)	0.319
	Use once a week	8 (22.2%)	11 (30.6%)	10 (27.8%)	7 (19.4%)	
	Use two to three times a week	1 (12.5%)	0 (0%)	2 (25.0%)	5 (62.5%)	
	Use three or more times a week	0 (0%)	1 (20.0%)	1 (20.0%)	3 (60.0%)	
	Use every day	0 (0%)	0 (0%)	2 (100%)	0 (0%)	
Dark Chocolate						
	Never consume	7 (13.7%)	17 (33.3%)	17 (33.3%)	10 (19.6%)	0.321
	Use once a week	17 (22.7%)	16 (21.3%)	21 (28.0%)	21 (28.0%)	
	Use two to three times a week	8 (21.1%)	10 (26.3%)	9 (23.7%)	11 (28.6%)	
	Use three or more times a week	4 (28.6%)	2 (14.3%)	1 (7.1%)	7 (50.0%)	
	Use every day	2 (50.0%)	0 (0%)	1 (25.0%)	1 (25.0%)	

*: p < 0.05.

Correlation analysis (Table 6) revealed that black coffee consumption was positively associated with expectations related to dependence, energy, mood, and physical performance. Black coffee consumption frequency showed a moderate positive correlation with dependence expectations (*r* = 0.549, *p* < 0.001) and weak to moderate correlation with energy, mood, and physical performance. Furthermore, mate tea consumption was positively correlated with sleep-related expectations; energy drink consumption was associated with perceived improvements in physical performance; and dark chocolate consumption was linked to expectations related to energy, physical performance, and sleep. The Spearman correlation coefficients indicated positive correlation of varying magnitudes (Table 6). These findings indicate that specific caffeinated products may be associated with different consumer expectations regarding their perceived effects.

**Table 6.** Spearman’s *rho* correlations between caffeine-containing food consumption frequency and caffeine-related expectations.

Variables		Consumption Frequency				
		Black Coffee	Mate Tea	Coca-Cola	Energy Drinks	Dark Chocolate
Consumption expectations	Dependence	*r* = 0.549	*r* = −0.036	*r* = 0.007	*r* = 0.089	*r* = 0.023
		*p* < 0.001*	*p* = 0.627	*p* = 0.931	*p* = 0.233	*p* = 0.754
	Energy	*r* = 0.303	*r* = 0.128	*r* = −0.048	*r* = 0.190	*r* = 0.177
		*p* < 0.001*	*p* = 0.084	*p* = 0.519	*p* = 0.010*	*p* = 0.017*
	Appetite	*r* = 0.111	*r* = 0.101	*r* = 0.110	*r* = 0.076	*r* = 0.141
		*p* = 0.135	*p* = 0.173	*p* = 0.138	*p* = 0.309	*p* = 0.058
	Mood	*r* = 0.380	*r* = 0.066	*r* = 0.006	*r* = 0.122	*r* = 0.115
		*p* < 0.001*	*p* = 0.375	*p* = 0.936	*p* = 0.102	*p* = 0.121
	Physical Performance	*r* = 0.292	*r* = 0.111	*r* = −0.052	*r* = 0.252	*r* = 0.168
		*p* < 0.001*	*p* = 0.136	*p* = 0.486	*p* < 0.001*	*p* = 0.024*
	Anxiety	*r* = 0.003	*r* = 0.115	*r* = −0.035	*r* = −0.009	*r* = 0.071
		*p* = 0.972	*p* = 0.121	*p* = 0.642	*p* = 0.903	*p* = 0.339
	Sleep	*r* = 0.101	*r* = 0.162	*r* = −0.043	*r* = 0.114	*r* = 0.170
		*p* = 0.173	*p* = 0.029*	*p* = 0.564	*p* = 0.126	*p* = 0.022*

r: Spearman’s rho correlation coefficient. Correlation strength was interpreted as weak (r < 0.30), moderate (0.30 ≤ r < 0.50), and strong (r ≥ 0.50). *: p < 0.05.

## 4. Discussion

The hypotheses proposed in this study were only partially supported. Although no direct association was found between overall caffeine consumption and oral behaviors or TMD symptoms, daily black coffee consumption was significantly associated with higher anxiety severity. Additionally, strong associations were observed between anxiety levels, oral behaviors, and the presence of painful TMD symptoms. These findings indicate that some, but not all, of the proposed associations were observed in the study sample.

The association between black coffee consumption and anxiety aligns with prior research indicating that caffeine may exacerbate anxiety symptoms by antagonizing adenosine A1 and A2A receptors, which regulate mood and arousal [11]. However, this mechanism should be interpreted as a theoretical framework, as the present study did not investigate the biological or genetic markers related to caffeine metabolism or receptor activity. The association between daily black coffee consumption and anxiety observed in the present study may contribute indirectly to TMD-related outcomes, since recent systematic reviews have identified anxiety as one of the main risk factors associated with temporomandibular disorders [8, 10].

The finding that only black coffee consumption, rather than total caffeine intake, was associated with anxiety symptoms may be explained by differences in consumption patterns and caffeine concentrations across products. Black coffee is often consumed in larger quantities and contains more caffeine per serving than other sources, such as chocolate or soft drinks. Additionally, contextual and behavioral factors related to coffee consumption may contribute to this association [11, 12].

The association between anxiety and oral behaviors found in this study may be understood within the framework of hypervigilance theory. This perspective suggests that individuals with heightened anxiety are more attuned to somatic sensations, potentially amplifying their perception of discomfort or pain [19, 20]. The well-established relationship between anxiety and TMD further substantiates this finding [8, 20].

Female participants demonstrated a higher prevalence of painful TMD and high-frequency oral behaviors. This finding is consistent with previous studies reporting a higher prevalence of painful TMD among women, which may be partially explained by biological, hormonal, and psychosocial factors. Recent evidence also suggests that anxiety may mediate the association between female sex and jaw function limitation in patients with TMD [4, 20].

Although a direct link between caffeine intake and TMD symptoms was not identified, the results suggest that psychological factors, particularly anxiety, may play an important role in the association between caffeine consumption, oral behaviors, and painful TMD symptoms [21].

Furthermore, the correlations observed between specific caffeinated products and expectations regarding their effects underscore the role of cognitive and behavioral factors in these consumption patterns. For example, the positive associations between black coffee and the expectancy domains related to dependence, mood enhancement, and physical performance reflect a usage profile frequently associated with habitual or compensatory consumption [22, 23]. Similarly, the associations between mate tea and sleep-related expectations and between energy drinks and physical performance reinforce the notion that perceived benefits guide caffeine use in function-specific contexts.

The association between dark chocolate consumption and expectations related to energy, sleep, and physical performance may reflect its potential stimulant properties and the psychological connotation of chocolate as an energizing and comforting food. However, the variability in caffeine metabolism driven by genetic and lifestyle factors complicates the interpretation of these correlations. Caffeine has a half-life of 2 to 10 h and can affect sleep quality depending on the timing of ingestion [13, 14], and sleep disturbances are known to negatively impact overall health and pain sensitivity [24].

Caffeine was also perceived as an ergogenic aid, particularly in the context of energy drink, black coffee, and chocolate consumption. This aligns with evidence from sports science, which has demonstrated the potential performance-enhancing effects of caffeine at low-to-moderate doses [25, 26].

Taken together, these findings should not be interpreted as evidence of causality, particularly given the cross-sectional and self-reported nature of this study. In addition, unmeasured factors such as sleep quality, academic stress, and the use of stimulant medications may have influenced these associations. Further research is needed to explore individual characteristics, such as psychological profiles, genetic predisposition, caffeine metabolism, or behavioral patterns, that may contribute to variations in these associations. Future studies would benefit from a longitudinal design and clinical assessment of TMD to validate and expand upon the present findings.

Among the limitations of this study, the cross-sectional design is important because it prevents the establishment of causal relationships. The findings should also be interpreted with caution, as the analyses were not adjusted for potential confounding factors such as sex, age, and other behavioral or psychosocial variables that may have influenced the observed associations. The recruitment strategy, highlighting the themes of “caffeine” and “TMD”, may have introduced selection bias by discouraging the participation of individuals who did not consume caffeine or exhibit TMD symptoms. Moreover, caffeine consumption was assessed using a non-validated, self-reported questionnaire based on intake frequency, without standardization into estimated total daily caffeine intake. Variations in caffeine content across different products may have influenced the comparability of results across caffeine sources. Additionally, the lack of clinical assessment for TMD diagnosis and the predominance of female participants may have influenced the results, despite the analytical approach adopted. Finally, the use of self-reported questionnaires may limit the robustness of the findings, as clinical confirmation would strengthen diagnostic accuracy. It is also important to note that no formal correction for multiple comparisons was performed. Therefore, the possibility of type I error cannot be excluded, and the findings should be interpreted with caution.

## 5. Conclusions

This study identified associations between daily black coffee consumption and severe anxiety, as well as anxiety, oral behaviors, and painful TMD symptoms in university students. Although overall caffeine intake was not directly associated with TMD or oral behaviors, daily black coffee intake was more frequently observed among participants with severe anxiety symptoms.

Additionally, distinct patterns emerged between specific caffeinated products and expectations regarding their perceived effects, highlighting the role of cognitive and behavioral factors in caffeine consumption patterns. These findings suggest a possible association between black coffee consumption, anxiety symptoms, and painful TMD-related outcomes; however, causality cannot be established because of the observational design of the study.

Future studies using more robust designs, biological assessments, and clinical TMD evaluations are recommended to further investigate the mechanisms underlying these associations and factors contributing to individual differences in caffeine-related responses.
